# Effects of ipratropium bromide on the occurrence of postoperative respiratory complications in craniectomy patients with COPD: A nationwide multicenter retrospective study

**DOI:** 10.1097/MD.0000000000020836

**Published:** 2020-06-26

**Authors:** Zhuoying Du, Xiaoqian Huang, Yi Feng, Wei Yan, Dan Xu, Xiaoou Sun, Chao Wu, Yongke Zheng, Longhuan Zeng, Xiaowei Xiong, Yuankun Liu, Chenbo Zhang, Jianfeng Luo, Jin Hu

**Affiliations:** a Department of Neurosurgery, Huashan Hospital of Fudan University; b Department of Biostatistics, School of Public Health, Fudan University; c NHC Key Laboratory of Health Technology Assessment (Fudan University); d Key Laboratory of Public Health Safety of Ministry of Education (Fudan University), Shanghai; e The Third Affiliated Hospital of Soochow University, Changzhou, Jiangsu; f The Second Affiliated Hospital, School of Medicine, Zhejiang University, Hangzhou, Zhejiang; g The First Affiliated Hospital of Chongqing Medical University, Chongqing; h The First Affiliated Hospital of Soochow University, Suzhou, Jiangsu; i Affiliated Hangzhou First People's Hospital Zhejiang University School of Medicine, Hangzhou, Zhejiang; j Wu Xi People's Hospital, Wuxi, Jiangsu, China.

**Keywords:** elective neurosurgical craniotomy, ipratropium bromide, postoperative pulmonary complications

## Abstract

**Introduction:**

Postoperative pulmonary complications (PPCs) are common and associated with increased morbidity, mortality, and medical cost. They are gaining increasing concerns among patients receiving neurological surgery. Chronic obstructive pulmonary disease (COPD) affect a large section of whole population and is also one of the risk factors of PPCs in the perioperative setting. Ipratropium bromide is the inhalation solution for the treatment of COPD. Studies showed the perioperative nebulization of ipratropium bromide could increase the lung function and decrease the incidence of postoperative pneumonia in COPD patients underwent thoracic surgery. The purpose of this study is to investigate the effect of perioperative nebulization of ipratropium bromide on PPCs in COPD patients underwent neurosurgical surgery.

**Methods and analysis:**

This study is a multicenter retrospective study in China. Patients who meet the inclusion/exclusion criteria are selected from 7 neurosurgical centers in China. According to whether ipratropium bromide is used in perioperative period, the patients are divided into exposure group and control group. The primary outcome is the incidence of postoperative pneumonia. Secondary outcomes are unplanned intubation, postoperative mechanical ventilation ≥ 48 hours, respiratory failure, atelectasis, death, and length of stay.

**Ethics and dissemination:**

This study was approved by the ethics committee (EC) of the School of Public Health, Fudan University, Shanghai, China. Waived by the ethics committee, no written consent form was obtained since we used the registry data. The study results will be communicated via publication.

**Trial Registration Number:**

ChiCTR1900022552.

## 1 Introduction

Postoperative pulmonary complications (PPCs), including postoperative pneumonia, atelectasis, and respiratory failure, are important risk factors with negative effects on the prognosis of surgical patients.^[1,2]^ Neurosurgical craniotomy related lung compression, long-term mechanical ventilation, and alteration of consciousness may associated with increased risk of PPCs.^[3–7]^ The American Academy of surgeons reviewed 38284 patients underwent craniotomy between 2005 and 2015. The incidence of all PPCs (including pneumonia, unplanned intubation, mechanical ventilation >48 hours, and pulmonary embolism) was 11.3%, and the incidence of postoperative pneumonia was 3.5%.^[8]^ Meanwhile, some studies have shown that operation time ≥300 minutes, postoperative mechanical ventilation ≥48 hours, intensive care unit (ICU) hospitalization time >3 days, postoperative low consciousness, chronic lung disease are independent risk factors for PPC.^[4]^ PPCs lead to the higher mortality, longer length of stay in both hospital and ICU, dramatic increase in medical cost and socioeconomic burden.^[1,7,9,10]^ Moreover, impaired pulmonary function, which is a remarkable manifestation of chronic obstructive pulmonary disease (COPD), is also one of the risk factors of PPCs.^[11–13]^ Some studies have confirmed that the introduction of COPD treatment can reduce the risk of PPCs.^[12]^ On the other hand, PPCs and their treatment can alter the disease course significantly in COPD patients.^[11–14]^ Therefore, it is important to take proper measures to reduce the incidence of PPCs to improve the quality of surgical care and reduce the medical cost.

Ipratropium bromide is a short acting bronchodilator, which can relax the airway smooth muscle and reduce the production of sputum. It can also improve the lung function of COPD patients by nebulized inhalation.^[5,15–17]^ In addition, perioperative use of ipratropium bromide can reduce the incidence of postoperative pneumonia and other PPCs in patients underwent abdominal and pulmonary surgery.^[18–20]^ Studies have shown that the perioperative use of ipratropium bromide was effective in prevention of PPCs in patients with severe head injury.^[21,22]^ However, the effect of inhaled ipratropium bromide on PPCs in COPD patients underwent elective neurosurgical craniotomy is not clear. Therefore, it is important and urgent to evaluate the effect of nebulization of ipratropium bromide on the incidence of PPCs in COPD patients underwent craniotomy.

## 2 Method and design

### 2.1 Hypothesis

Perioperative use of ipratropium bromide can reduce the incidence of PPCs in COPD patients underwent elective neurosurgical craniotomy.

### 2.2 Design

The study is a multicenter retrospective study. Patients who meet the inclusion criteria will be included from the neurosurgery department of 7 medical centers in China. According to whether ipratropium bromide was used in perioperative period, the patients will be divided into exposure group and control group. Data of perioperative period and hospitalization period will be collected from patients’ medical records using structured questionnaire by trained neurosurgeon at each medical center. Eight hundred thirty-four patients will be enrolled in this study.

**Figure 1:**
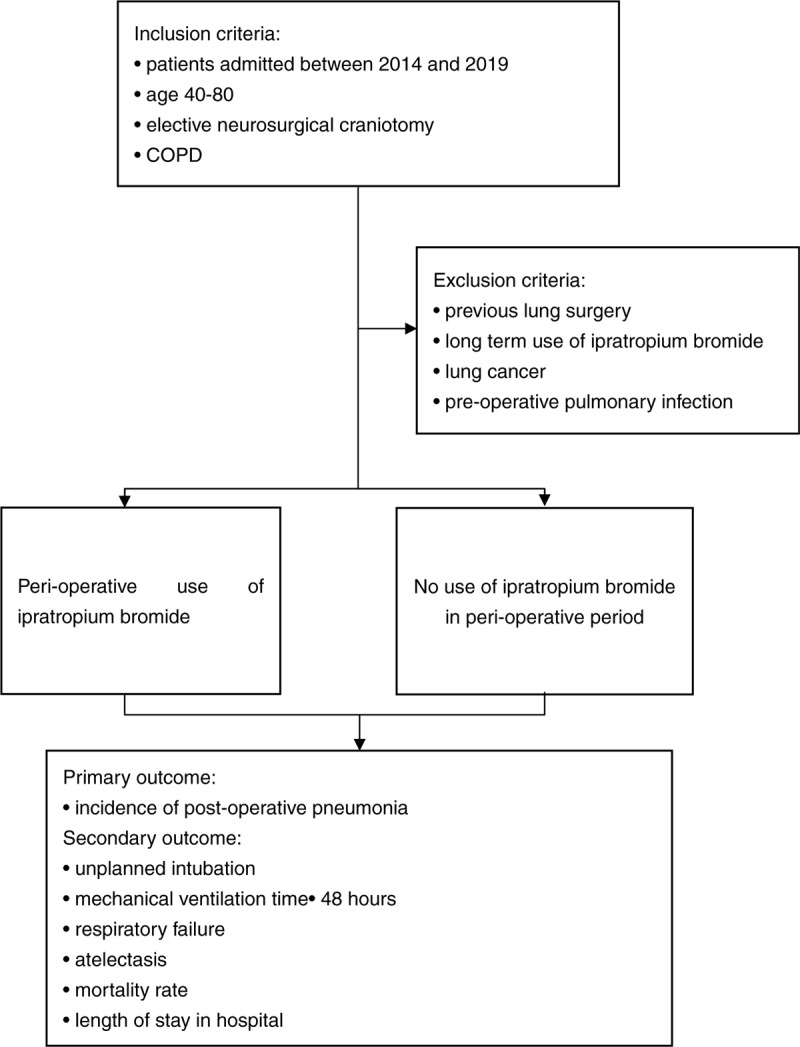
Flow chart of the study. COPD = chronic obstructive pulmonary disease.

The study arrangement was as the following flow chart (Fig. 1).

### 2.3 Research purposes

#### 2.3.1 Main purpose

To evaluate the effect of ipratropium bromide on the incidence of postoperative pneumonia in patients with COPD and underwent elective neurosurgical craniotomy.

#### 2.3.2 Secondary purposes

To evaluate the effect of ipratropium bromide on

(1) The incidence of pulmonary complications in patients with COPD and underwent elective neurosurgical craniotomy.
(2) The length of stay in patients with COPD and underwent elective neurosurgical craniotomy.
(3) The postoperative mortality in patients with COPD and underwent elective neurosurgical craniotomy.

### 2.4 Study population

Patients with COPD and underwent elective neurosurgical craniotomy from 2014 to 2019 in 7 neurosurgical centers in mainland China.

#### 2.4.1 Setting

The participating centers includes neurosurgery departments in Huashan Hospital of Fudan University, Wu Xi People's Hospital, Hang Zhou First People's Hospital, The First Affiliated Hospital of Chongqing Medical University, The First Affiliated Hospital of Soochow University, The Third Affiliated Hospital of Soochow University and The Second Affiliated Hospital of Zhejiang University. All of these neurosurgery centers will assign a coordinator and a neurosurgeon to participate in this study. The neurosurgeon will take the responsibility to collect data from medical record. Consistent and strict inclusion criteria will be applied to reduce selection bias and ensure the quality.

#### 2.4.2 Inclusion criteria

(1) Aged 40 to 80 years
(2) Underwent elective neurosurgical craniotomy
(3) Patients have any of the following lung conditions:
  - Forced expiratory volume in 1 second/forced vital capacity <70% in preoperative pulmonary function examination
  - Emphysema in pulmonary imaging
  - History of COPD, chronic bronchitis and emphysema

#### 2.4.3 Exclusion criteria

(1) Patients with previous lung operation
(2) Regular use of ipratropium bromide before admission
(3) Patients with lung cancer
(4) Lung infection before operation

### 2.5 Exposure and control

#### 2.5.1 Exposure

The use of ipratropium bromide inhalation solution during perioperative period (3 days before operation to 7 days after operation).

#### 2.5.2 Control

Without use of ipratropium bromide inhalation solution during perioperative period.

### 2.6 Study covariates

#### 2.6.1 Preoperative variables

(1) Basic characteristics of patients
  - Demographic characteristics: age, gender, height, weight
  - Pulmonary function: forced expiratory volume in 1 second/forced vital capacity
  - Smoking history
  - Location of the lesion: supratentorial convex, posterior skull base, anterior skull base, middle skull base, brain stem, hypothalamus pituitary third ventricle, lateral ventricle, fourth ventricle
  - Body position during operation: supine, prone, lateral
  - Operation type: direct vision operation, microsurgery, endoscopic operation
(2) Major complications
  - Cardiovascular: arrhythmia, hypertension
  - Respiratory diseases: asthma, sleep apnea syndrome
  - Renal function: serum creatinine value, urea nitrogen value
  - Endocrine system diseases: diabetes (blood glucose level)
(3) Preoperative medication including the ipratropium bromide and the related pre-operative medication (including expectorant, inhaled corticosteroid (ICS), long-acting muscarinic antagonist (LAMA), long-acting β_2_-agonist (LABA)/LAMA, ICS/LABA, theophylline)

#### 2.6.2 Intraoperative variables

The operation related time is as follows:

(1) Operation time: from the beginning to the end of the operation
(2) Anesthesia time: from induction to extubation
(3) Extubation time: from the end of operation to the extubation of endotracheal tube
(4) Length of stay in neurologic intensive care unit (NICU): From entering post anesthesia care unit (PACU) to preparing to transfer out PACU

#### 2.6.3 Postoperative variables

(1) Postoperative medication: ipratropium bromide, antibiotic, other related postoperative medication (including expectorant, ICS, LAMA, LABA/LAMA, ICS/LABA, theophylline)
(2) Other postoperative complications: consciousness disorder (Glasgow coma scale <8), limb paralysis, aspiration

#### 2.6.4 Post discharge variables

The use of medical resources and medical expenses are as follows:

(1) The length of stay in ICU
(2) The length of stay in hospital
(3) The total medical cost

### 2.7 Study endpoints

#### 2.7.1 Primary outcome

The primary outcome is the incidence of postoperative pneumonia in different groups. The diagnostic criteria of postoperative pneumonia are new or progressive infiltrative lesions on chest imaging, with the following 2 or more symptoms: body temperature ≥38.5°C or <36°C, peripheral blood leukocyte >12 × 10^9^ / L or <4 × 10^9^ / L, purulent sputum and/or new or aggravated cough and expectoration.

#### 2.7.2 Secondary outcome

(1) Atelectasis. The manifestation of atelectasis indicated by chest imaging.
(2) Respiratory failure. The diagnostic criteria of respiratory failure are PaO_2_ <60 mm Hg or SpO_2_ <90%, or PaO_2_ /FiO_2_ ≤ FIO under the condition of oxygen inhalation
(3) Unplanned intubation. It means unplanned re-intubation after operation
(4) Mechanical ventilation time ≥48 hours
(5) Length of stay in hospital
(6) Mortality rate

### 2.8 Ethics

This study was approved by the Ethics Committee of the School of Public Health, Fudan University, Shanghai, China. Waived by the approving EC, no written consent form was obtained since we used the registry data.

### 2.9 Study monitoring

Inclusion and exclusion criteria will be reviewed by the investigator to ensure that participants meet the study criteria. The school of public health of Fudan University will be responsible for research supervision, schedule management, data management, and statistical analysis.

### 2.10 Access to data

The data collected in the study will be recorded in electronic case report form by researchers, including the basic characteristics of patients, pulmonary function, location of tumor, body position during operation, operation type, major complications, preoperative medication, operation time, anesthesia time, extubation time, PACU stay time, PPCs, other clinical complications, hospitalization time, ICU time, hospitalization cost, death, and major related postoperative medication.

### 2.11 Statistical analysis plan

The statistical analysis plan will be completed before the database is locked and confirmed by the main researchers and the sponsor. The statistical methods used will be explained more comprehensively in the data analysis plan, including the rules and data processing agreements when the analysis is conducted, and the methods used to process the missing data. Any change plan will appear as attachment before database locking, and any change after database locking will be recorded in clinical research report.

### 2.12 Sample size calculation

According to the study results in the published literature, the incidence of postoperative pneumonia was 7.5% in the ipratropium bromide treatment group and 15% in the control group. 80% of the test efficacy (1-β) and 5% of the test level (α) are given. The proportion of the treatment group and the control group is set at 1:1. The sample size required for the 2 groups is 278 cases, respectively. With the possible loss rate of 33% in the process of propensity score match (PSM), 417 samples will be needed in each group, a total of 834.

### 2.13 Statistical methods

Continuous variables will be represented by mean and standard deviation or median and interquartile range. Categorized variables will be represented by percentage (%), Student *t* test, ANOVA, Wilcoxon rank sum test, Pearson Chi square test, Fisher exact probability method, and Cochran–Mantel–Haensel Chi-square test will be used to analyze the baseline characteristics and end-point of patients.

The primary outcome is the incidence of postoperative pneumonia. Logistic regression analysis will be used for multivariate analysis. Considering the imbalance of covariates in non-randomized research, the PSM will be used to reduce the impact of the differences between the 2 groups on the end of the study. The variables included in PSM will be determined by the results of univariate analysis and clinical conditions. The statistical analysis method of secondary outcomes will be the same as that of primary outcome. The length of stay will be analyzed by linear model with log transformation. All analyses will be performed using SAS 9.4 (*P* < .05).

## 3 Discussion

PPCs are the most frequently reported postoperative complications after surgery.^[7,23,24]^ PPCs will increase the length of stay of patients and increase the consumption of medical resources.^[20]^ There have been several studies reported the disease burden and risk factors of PPCs in neurosurgery, which are the fundamental for the identification of effective interventions for PPCs prevention.^[1,6]^ Until now, the enhanced recovery after surgery concept is well adopted and implemented in general surgery and neurosurgery care, which is proved effective in decrease of complications and length of hospital stay.^[25,26]^ To our knowledge, this is the first study to investigate the drug intervention on the rates of PPCs in COPD patients underwent elective intracranial surgery, which may further provide evidence on the enhanced recovery after surgery in Neurosurgery.

PPCs are well described in patients receiving intracranial surgery. Ideal positioning of head and body for surgery often means extra stretching or compression of the airway, which can cause the pressure of airway to rise and subsequent mechanic injury. In addition, longer anesthesia and ventilation time are frequently needed due to the complexity of neurosurgical procedures, which will result in not only increased risks of airway spasm and mechanic injury, but also higher rates of drug-related postoperative sickness and vomiting.^[27–30]^ The latter are known risks for pulmonary inspiration and infection. Moreover, surgery for lesions in the posterior cranial fossa may affect the respiratory center and cause impaired ventilation, even mechanic ventilation dependence.^[31]^ Other complications of neurosurgery, such as ischemia and other neurological deficits, are also associated with risks of altered consciousness, delayed extubation as well as unexpected reintubation after surgery.

Pneumonia is one of the most common form of PPCs after intracranial surgery. COPD is characterized by conditions such as airway hypersensitivity, increased production of sputum, and impaired ventilation. Given the surgical aspects, COPD is definitely a risk factor of PPCs in surgery patients.^[32]^ In China, COPD affected 13.7% of people over 40 years of age and results in a hospital stay rate of 1.6% in 2015.^[33,34]^ COPD is characterized by persistent respiratory symptoms and airflow limitation that is due to airway and/or alveolar abnormalities. Therefore, it is very important to reduce PPC for patients underwent craniotomy, especially those with COPD. Physical therapies such as cessation of smoking and respiratory training have been proposed as preoperative preparation measures. Short-term bronchodilators, like ipratropium bromide might improve the lung function of patients underwent craniotomy, hence decrease the risk of PPC.^[35,36]^

With careful research design, this study can provide useful information for preventive strategies for PPCs after intracranial surgery. As the most common form of PPCs, the incidence of postoperative pneumonia as selected as the primary outcome. The incidence of other pulmonary complications, length of stay, and postoperative mortality are secondary outcomes. Previous lung surgery, use of ipratropium bromide before admission, previous lung cancer, and preoperative lung infection will affect the analysis of drug efficacy in this study, so they are used as exclusion criteria. Although it will impact the generalization of the results of this study, the exclusion criteria will decrease the heterogeneity of subjects and consistence of this study. The inhalation of ipratropium bromide may decrease the risk of PPCs through relaxing the airway smooth muscle and reducing the production of sputum not only during but also after the operation. Six out of 7 medical centers in this study locates in eastern China around Shanghai, with the First Affiliated Hospital of Chongqing Medical University locates in western China. It was the balance of representative and convenience.

This study has several limits. First of all, common PPCs does not have a consistent definition at present, so we used the definitions adopted by most of the studies, which is pneumonia, atelectasis, respiratory failure, unplanned intubation, postoperative mechanical ventilation ≥48 hours.^[6,12,37–39]^ Second, it is possible that the definition of a symptom or disease changes over time. Fortunately, the study subjects are COPD patients underwent craniotomy from 2014 to 2019. Within this time span, little change has happened in medical diagnosis and treatment techniques. Third, physicians in different hospitals may adopt inconsistent diagnosis criteria and treatment protocol. The coordinator and neurosurgeon in each participated hospital was trained to make sure they understood the protocol and be consistent on data collection. The sample size of exposure and control group were required to be 1:1 in each participated hospital to decrease the impact of the heterogeneity of hospital. A pilot visit to each hospital has been conducted to make sure the feasibility and consistence of data collection. We will collect a quite large number of covariates and use multivariate analysis and PSM methods in the data statistical phase to control potential bias. Finally, the study is retrospective based on case registration. The shortcomings of retrospective research can’t be ruled out in our study.

### 3.1 Trial status

This study is registered in CHiCTR and the trial number is ChiCTR1900022552. In February 2019, retrospective collection of medical records of the first patient was started. The retrospective collection of the medical records of the last patient will be completed in November 2020. The research report is expected to be completed in September 2021. The study results will be communicated via publication.

## Author contributions

JL and JH conceived and designed the study, ZD and XH wrote the manuscript. YF, WY, DX, XS, CW, YZ, LZ, XX, YL, CZ acquired the data and managed the trial at each of the sites. All authors were involved in critical appraisal and revision of the manuscript. All authors approved the final manuscript prior to submission.

**Conceptualization:** Jianfeng Luo, Jin Hu.

**Writing – original draft:** Zhuoying Du, Xiaoqian Huang.

**Writing – review & editing:** Zhuoying Du, Xiaoqian Huang, Jianfeng Luo, Jin Hu.
